# 慢性髓细胞性白血病慢性期患者二次无治疗缓解6例报告并文献复习

**DOI:** 10.3760/cma.j.cn121090-20250803-00359

**Published:** 2026-01

**Authors:** 天骄 黄, 莹 武, 岩 尉, 晓甦 赵, 亚溱 秦, 红霞 石, 晓军 黄, 浩 江

**Affiliations:** 北京大学人民医院、北京大学血液病研究所，北京 100044 Beijing University People's Hospital,Peking University Institute of Haematology, Beijing 100044, China

## Abstract

慢性髓细胞性白血病慢性期（CML-CP）患者二次尝试无治疗缓解（TFR）缺乏临床数据和推荐方案。本文回顾分析2006年10月至2024年10月北京大学人民医院诊治的二次酪氨酸激酶抑制剂（TKI）停药尝试TFR的6例CML-CP患者的临床资料。6例患者首次TKI停药前均接受中位82.5（40～87）个月的伊马替尼治疗，持续深层分子学反应即达分子学反应4.5（MR^4.5^）的中位时间为34.5（24～62）个月，于停用伊马替尼中位3（2～4）个月时失去主要分子反应（MMR）。6例患者均在第1次TFR（TFR1）失败后4周内重启TKI治疗，6例患者接受重启TKI治疗后中位4.5（3～7）个月再次获得MR^4.5^，并于重启TKI后93、84、76、76、33、44个月自愿尝试第二次TKI停药，4例患者仍TFR，持续TFR时间分别为42、23、21及58个月。重启TKI治疗时间大于6年、持续MR^4.5^大于5年的4例患者均获得≥21个月持续TFR2，其中3例重启TKI治疗时应用过1年以上的二代TKI。另2例于二次TKI停药后4和3个月失去MMR，再次重启TKI后2和3个月后重获MR^4.5^。

## Abstract (en)

Data and guidelines for a second attempt at treatment-free remission（TFR）in patients with chronic myeloid leukemia in the chronic phase（CML-CP）are limited. We retrospectively analyzed the clinical data of six patients with CML-CP who attempted a second discontinuation of tyrosine kinase inhibitors（TKIs）for TFR at Peking University People's Hospital from October 2006 to October 2024. Before the first TKI discontinuation, all six patients had received imatinib treatment for a median of 82.5 months（range, 40–87 months）and maintained a sustained deep molecular response, specifically MR^4.5^, for a median of 34.5 months（range, 24–62 months）. They lost major molecular response（MMR）at a median of 3 months（range, 2–4 months）after discontinuing imatinib treatment. All six patients resumed TKI treatment within 4 weeks following failure of the first TFR（TFR1）. After a median of 4.5 months（range, 3–7 months）on TKI, they achieved MR^4.5^ again and voluntarily attempted a second TKI discontinuation at 93, 84, 76, 76, 33, and 44 months, respectively, after resuming therapy. Four out of six patients maintained TFR, with sustained TFR durations of 42, 23, 21, and 58 months, respectively. All four patients who had resumed TKI treatment for >6 years and maintained MR^4.5^ for >5 years achieved a sustained second TFR of ≥21 months; among these, three had received second-generation TKI for >1 year during the resumption of TKI treatment. The other two patients lost MMR at 4 and 3 months, respectively, after the second TKI discontinuation but regained MR^4.5^ at 2 and 3 months, respectively, after resuming TKI treatment again.

酪氨酸激酶抑制剂（TKI）的问世显著改善慢性髓细胞性白血病慢性期（CML-CP）患者生存，然而新的医学问题，如晚期不良事件（心脑血管事件和肾功能障碍等）以及由于长期用药而导致的高额费用等问题日渐影响患者的生存质量。

多项研究证实[1]–[8]，CML-CP患者TKI停药获得持久性无治疗缓解（TFR）是可行的。TKI停药获得TFR已成为CML-CP患者的最终治疗目标，并写入中外指南[9]–[10]。IRIS研究中[11]，一代TKI治疗随访2年以上累计25％患者达到分子学反应4.5（MR^4.5^），ENESTnd[12]和DASISION[13]研究中，二代TKI治疗随访终点5年累计53％和42％患者达到MR^4.5^，故总体仅有15％～30％的CML慢性期患者能够成功获得TFR。失去TFR的患者重启TKI治疗后绝大部分能够短期内重获MR^4.5^，并持续维持深层分子学反应（DMR）。为提高CML整体TFR比例，近年来国外一些研究报道了即使第1次TFR（TFR1）失败后，仍能成功实现TFR2，国内尚无相关研究报道。我们报道北京大学血液病研究所二次TKI停药尝试成功实现TFR2的6例CML-CP患者。

## 病例与方法

1. 研究对象：纳入2006年10月至2024年10月就诊北京大学人民医院门诊CML-CP患者中应用伊马替尼治疗3年以上同时达DMR 2年以上尝试第1次停药，停药后失去主要分子反应（MMR）即分子学复发，分子学复发后再次接受TKI治疗后达到DMR，进行第2次尝试停药的6例患者，随访截止时间2025年6月30日。

2. 分子学反应和分子学复发定义：依据欧洲白血病网（ELN）标准[14]，MMR定义为国际标准化（IS）BCR::ABL1≤0.1％。MR^4.0^定义为BCR::ABL1^IS^≤0.01％，MR^4.5^定义为BCR::ABL1 ^IS^≤0.0032％，MR^5.0^定义为BCR::ABL1 ^IS^≤0.001％。DMR定义为MR^4.0^或更深程度缓解。分子学复发定义为两次连续检测失去DMR或1次检测失去MMR。

3. 停药标准及随访时间：根据美国国家癌症综合网络（NCCN）指南[9]及CML中国诊断与治疗指南[10]成人CML停药标准：①年龄≥18周岁；②CML-CP患者，无加速期（AP）及急变期（BP）既往史；③接受TKI治疗≥3年；④既往有可定量的BCR::ABL1转录证据；⑤稳定DMR≥2年,至少4次监测记录，每次间隔≥3个月；⑥获得可靠的定量（qPCR）检测，检测灵敏度至少为MR^4.5^，并在2周内提供结果；⑦停药后前6个月内每1～2个月进行1次分子学监测，第7～12个月期间每2个月进行1次，此后每季度（无限期）对仍处于MMR的患者进行1次分子学监测；⑧MMR丧失后4周内立即恢复TKI治疗，每月进行1次分子学监测，直至MMR重新建立，对于MMR丧失后重新开始TKI治疗的患者，建议此后每3个月进行1次监测。如果在TKI恢复治疗3个月后未达到MMR，则应进行BCR::ABL1激酶结构域突变检测，并应继续每月进行1次分子学监测，持续6个月。

## 结果

患者首次及二次TKI停药临床信息详见表1、表2。

**表1:** 6例慢性髓细胞性白血病慢性期患者首次停药时基本临床资料

例号	性别	初诊年龄（岁）	既往疾病	Sokal评分	首次TKI治疗种类	用药剂量（mg/d）	是否自行停药	MR^4.5^持续时间（月）	停药前TKI治疗时间（月）	药物不良反应	首次停药到失去MMR时间（月）
1	男	49	/	中危	IM	400	否	36	61	水肿、肌酐高	2
2	女	33	/	低危	IM	400	否	33	80	骨痛	3
3	男	45	乙肝携带	低危	IM	400	否	41	85	水肿、骨痛	4
4	女	43	/	低危	IM	400	否	62	85	水肿	3
5	女	12	/	高危	IM	400	否	24	87	水肿、骨痛	3
6	男	51	/	中危	IM	400	否	26	40	/	4

**注** TKI：酪氨酸激酶抑制剂；MR^4.5^：分子学反应4.5；MMR：主要分子学反应；IM：伊马替尼；/：无疾病或药物不良反应

**表2:** 6例慢性髓细胞性白血病慢性期患者第二次停药临床资料

例号	再次服药时年龄（岁）	再次TKI治疗种类/治疗时间（月）/不良反应	是否更换TKI	更换TKI治疗种类/治疗时间（月）/不良反应	重启TKI至第二次尝试停药前时间（月）	重启TKI治疗后达MR^4.5^时间（月）	MR^4.5^持续时间（月）	是否自行停药	TFR2时间（月）	第二次停药后随访时间是否失去MMR
1	55	IM/81/无	是	DA/12/无	93	4	89	否	42	否
2	40	IM/42/无	是	NIL/42/无	84	5	79	否	23	否
3	53	IM/56/无	是	NIL/20/脑梗死	76	6	70	否	21	否
4	50	IM/76/无	否	–	76	3	73	否	58	否
5	19	IM/33/无	否	–	33	7	26	是	4	是
6	55	IM/44/无	否	–	44	4	40	是	3	是

**注** TKI：酪氨酸激酶抑制剂；MR^4.5^:分子学反应4.5；MMR：主要分子学反应；IM：伊马替尼；DA：达沙替尼；NIL：尼洛替尼；–：不适用

例1，男，初诊年龄49岁，Sokal中危，首次停药前伊马替尼（400 mg/d）治疗61个月，持续达到MR^4.5^为36个月，服药期间先后出现水肿、肌酐高等不良反应，均可耐受，首次停药后2个月失去MMR。于TFR1失败4周内重启TKI治疗，重启TKI后接受伊马替尼（400 mg/d）81个月及达沙替尼（100 mg/d）治疗12个月。自愿尝试二次TKI停药，于重启TKI治疗4个月再次获得MR^4.5^，持续MR^4.5^时间89个月时，尝试二次TKI停药，两次停药期间均有停药不良反应（肌肉关节痛），至随访截止时42个月未失去MR^4.5^，即TFR2 ≥42个月。

例2，女，初诊年龄33岁，Sokal低危，首次停药前伊马替尼（400 mg/d）治疗80个月，持续达到MR^4.5^为33个月，服药期间出现骨痛等不良反应，可耐受，首次停药后3个月失去MMR。于TFR1失败4周内重启TKI治疗，重启TKI后接受伊马替尼（400 mg/d）42个月及尼洛替尼（600 mg/d）治疗42个月后自愿尝试二次TKI停药，重启TKI治疗5个月时再次获得MR^4.5^，重启TKI后持续MR^4.5^时间为79个月，两次停药期间均有停药不良反应（肌肉关节痛），至随访截止时23个月未失去MR^4.5^，即TFR2 ≥23个月。

例3，男，初诊年龄45岁，Sokal低危，首次停药前伊马替尼（400 mg/d）治疗85个月，持续达到MR^4.5^为41个月，服药期间先后出现水肿、骨痛等不良反应，均可耐受，首次停药后4个月失去MMR。于TFR1失败4周内重启TKI治疗，重启TKI治疗后6个月再次获得MR^4.5^，重启TKI后接受伊马替尼（400 mg/d）56个月及尼洛替尼（600 mg/d）20个月治疗，重启TKI后持续MR^4.5^ 70个月时，服用尼洛替尼期间出现脑梗死，在医师建议下尝试二次TKI停药，两次停药期间均无停药不良反应，至随访截止时21个月未失去MR^4.5^,即TFR2≥21个月。

例4，女，初诊年龄43岁，Sokal低危，首次停药前伊马替尼（400 mg/d）治疗85个月，持续达到MR^4.5^为62个月，服药期间出现水肿等不良反应，可耐受，首次停药后3个月失去MMR。于TFR1失败4周内重启伊马替尼治疗，重启伊马替尼治疗后3个月再次获得MR^4.5^，再次接受伊马替尼（400 mg/d）76个月治疗后自愿尝试二次TKI停药，此时再次持续MR^4.5^时间为73个月，两次停药期间均有停药不良反应（肌肉关节痛），二次TKI停药至随访截止时58个月未失去MR^4.5^，即TFR2 ≥58个月。

例5，女，初诊年龄12岁，Sokal高危，首次停药前伊马替尼（400 mg/d）治疗87个月，持续达到MR^4.5^为24个月，服药期间出现水肿、骨痛等不良反应，可耐受，首次停药后3个月失去MMR。于TFR1失败4周内重启TKI治疗，接受伊马替尼（400 mg/d）33个月治疗后因妊娠自行尝试二次TKI停药，重启TKI治疗后7个月即再次获得MR^4.5^，持续MR^4.5^时间为26个月，二次停药后4个月失去MMR，续用干扰素治疗，停药13个月后分娩，分娩2周后再次接受伊马替尼（400 mg/d）治疗，两次停药期间均无停药不良反应，第三次TKI治疗后2个月达MR^4.5^。

例6，男，初诊年龄51岁，Sokal中危，首次停药前伊马替尼（400 mg/d）治疗40个月，持续达到MR^4.5^为26个月，首次停药后4个月失去MMR。于TFR1失败4周内重启TKI治疗，重启TKI治疗后4个月即再次获得MR^4.5^，持续MR^4.5^时间为40个月时，即再次接受伊马替尼（400 mg/d）44个月后自行尝试二次TKI停药，二次停药3个月后失去MMR，4周内再次接受尼洛替尼（600 mg/d）治疗，两次停药期间均无停药不良反应，第三次TKI治疗后3个月达MR^4.5^。

## 讨论及文献复习

自从第一代TKI伊马替尼于1998年开始用于人体，并于2001年获得美国食品及药品管理局（FDA）批准治疗CML，开启了CML靶向治疗革命性进程，CML患者的生存期得到显著延长。应用TKI治疗CML-CP患者二十余年间，全球的血液学者们不断探索更新治疗策略，TFR已成为CML-CP患者的治疗目标。

STIM研究是一项发表于2010年的开创性TKI停药研究[1]，该研究通过对接受伊马替尼至少3年并维持DMR 2年的CML-CP患者尝试停药，1年TFR率为41％，2年TFR率为38％。A-STIM研究[2]是在STIM研究的基础上延长了TKI治疗及DMR维持时间，1年、2年及3年的TFR率分别为64％、64％和61％。似乎表明增加TKI治疗时间以及维持更长DMR时间有助于提高TFR率。欧洲终止酪氨酸激酶抑制剂（EURO-SKI）试验[3]是迄今为止规模最大的TKI停药试验，2年的TFR率为50％。二代TKI停药研究更新了达沙替尼、尼洛替尼停药数据，达沙替尼停药试验DADI[4]1年TFR率为48％，尼洛替尼停药试验ENEST freedom[5]和STAT2[6]1年TFR率分别为51.6％和67.9％。三代TKI停药研究补充了普纳替尼停药数据[7]，纳入11例在停药前接受普纳替尼治疗并达DMR的CML-CP患者，1年及2年TFR率分别为64％和53％。二代TKI和三代TKI停药研究TFR率高于一代TKI停药研究，显示对BCR::ABL1更强效的TKI有助于提高TFR率。

RE-STIM研究是首个TKI再次停药（TFR2）研究[15],该研究纳入70例经过TFR1失败后重启TKI治疗并达到DMR的CML-CP患者。他们尝试TFR2前再次接受中位时间32个月TKI（其中50例伊马替尼，7例达沙替尼及13例尼洛替尼）治疗并维持中位时间为25个月的DMR，1年、2年及3年的TFR2率为48％、42％和35％。该研究首次证明TFR2尝试是安全的，且TFR1失败并不排除TFR2成功停药的可能性。A-STIM研究[16]纳入32例经过TFR1失败后再次尝试TFR2的CML-CP患者，1年和3年TFR2率为46.8％和35.8％。Nilo post-STIM研究[17]纳入22例尝试伊马替尼停药失败的CML-CP患者，再次接受尼洛替尼治疗至少2年并维持中位时间22个月的DMR，1年、2年和3年的TFR2率分别为68.2％、59.1％和54.2％。DAstop2研究[18]纳入62例尝试任何TKI治疗失败的CML-CP患者，再次接受中位时间65个月达沙替尼治疗并维持DMR中位时间59个月，1年和2年的TFR2率分别为56％和46％。这些数据表明二代TKI治疗在第2次停药亦能达到50％左右的TFR2率（表3）。

**表3:** 文献报道TFR2的慢性髓细胞性白血病慢性期患者临床资料

文献来源	国家	发表年份	第二次停药患者例数	首次治疗TKI类型	首次TKI治疗中位时间	首次TKI停药前DMR中位时间	重启治疗TKI类型	重启TKI治疗中位时间	重启TKI至再次停药前DMR中位时间
Legros[15]	法国多中心	2017	70	IM/DA/NIL	59个月	32个月	IM/DA/NIL	32个月	25个月
Rousselot[16]	法国多中心	2020	32	IM/二代TKI	6年	NR	IM/二代TKI	3.4年	2.3年
Ureshino[22]	日本单中心	2021	10	IM/DA/NIL	40.1个月	27.8个月	DA/NIL	36.9个月	34个月
Kim[20]	韩国	2022	21	IM	68个月	32.9个月	IM	31个月	5.6个月
Dulucq[17]	法国多中心	2023	22	IM	NR	NR	NIL	≥2年	22个月
Flygt[18]	欧洲多中心	2024	62	任何TKI	NR	NR	DA	65个月	59个月

**Table:** 

文献来源	第二次停药后随访中位时间	1年TFR2率［％（95％ *CI*）］	2年TFR2率［％（95％ *CI*）］	3年TFR2率［％（95％ *CI*）］
Legros[15]	38.3个月	48（37.6～61.5）	42（31.5～55.4）	35（24.4～49.4）
Rousselot[16]	4.9年	46.8（29.1～62.7）	NR	35.8（52.5～19.4）
Ureshino[22]	32.3个月	37.5（9.9～65.9）	NR	NR
Kim[20]	NR	NR	NR	NR
Dulucq[17]	52个月	68.2（51.3～90.7）	59.1（41.7～83.7）	52.4（36.8～79.8）
Flygt[18]	27个月	56（44～70）	46（36～62）	NR

**注** TFR2：第2次无治疗缓解；TKI：酪氨酸激酶抑制剂；DMR：深层分子学反应；NR：未提及；IM：伊马替尼；NIL：尼洛替尼；DA：达沙替尼

我国首次多中心回顾性研究[19]报道CML-CP患者自动停药TFR率为58.2％，与国外报道相当，且停药前MMR维持时间越长，停药后复发率越低。我中心的前期研究[8]表明伊马替尼治疗持续时间>85个月以及MR^4.5^持续时间>54个月与成功TFR相关，停药后接受干扰素治疗有助于TFR，而患者的年龄、性别、Sokal评分和达到MR^4.5^的时间与TFR无关。本研究报道6例患者均在TFR1失败4周内再次接受TKI治疗，重启TKI累计时间大于6年，持续MR^4.5^大于5年的4例患者均获得TFR2大于21个月，其中3例重启治疗应用过1年以上的二代TKI。例5及例6重启TKI伊马替尼仅33及44个月（因妊娠、自行停药），尽管再次MR^4.5^持续24个月及40个月，TFR2失去MMR。尝试TFR2前巩固阶段的最佳持续时间尚不清楚，Kim等[20]报道真实世界TFR2尝试并应用数学模型预测TFR2前最佳伊马替尼治疗时间为5年，而本研究4例成功TFR2前再次接受6年以上的TKI治疗且持续MR^4.5^超过5年。其中1例TFR2前再次接受伊马替尼巩固治疗，成功停药随访58个月，提示伊马替尼耐受性良好，可作为TFR2尝试前的巩固治疗，而有报道表明患者对尼洛替尼或达沙替尼的耐受性较低，并可能因药物相关不良事件导致停药[17]。虽然目前没有证据支持将伊马替尼改为达沙替尼或尼洛替尼作为TFR2前治疗，但本研究3例TFR2前接受达沙替尼或尼洛替尼治疗均成功停药随访超过1年，说明达沙替尼或尼洛替尼亦可能成为TFR2前治疗的选择之一。日本学者曾报道持续服用伊马替尼5年，所有患者均出现不同程度肾功能减退，在尝试TFR的患者中发现，停药1年后大部分患者肾功能不全可逆性恢复[21]。本研究3例TFR2前更换二代TKI可以规避伊马替尼的肾脏损害。本研究为中国CML患者单中心TFR2首次报道，病例数尚少，结合2017年以来国际上数个中心共200余例CML患者TFR2的报道（表3），多数报道在尝试TFR2前患者应用了二代TKI，TFR2成败分析受病例数少及各种因素限制。随着医师与患者对TFR安全性及必要性的认识提高，有较大空间可扩大病例数支持进一步研究。DAstop2研究[18]报道在首次停药后3个月内出现分子学复发的患者6个月的TFR2率仅为13％。RE-STIM研究[15]观察到首次停药后3个月时仍保持MMR的患者TFR2率更高。而Nilo post-STIM研究[17]未发现首次和第二次分子学复发的时间之间有相关性。另一项来自日本真实世界报道4例成功TFR2患者均为男性[22]，提示性别可能与TFR2相关，本研究报道4例成功TFR2患者为2例男性和2例女性。另外多项研究报道约10％患者TFR晚期分子学复发[15]–[16],故本研究仍需长期随访，进一步探究TFR2成功相关因素。

ELN基于既往发表研究数据制定最新CML-CP患者停药指导意见[23]，建议TFR1最佳停药时间为TKI治疗持续时间超过5年，比既往推荐延长2年，同时MR^4.0^持续时间超过3年或MR^4.5^持续时间超过2年，同时建议增加停药后分子学监测频率。

TFR已成为TKI治疗CML的终极目标，中位25％的CML-CP患者可获得TFR1。真实世界中CML-CP患者的TFR2安全可行，TFR2前长期TKI巩固治疗和维持MR^4.5^及二代TKI似乎有助于TFR2成功。TFR2的成功可提高CML人群的总体TFR比例至35％～40％。已有报道第三次无治疗缓解（TFR3）成功案例[23]。CML-CP初始治疗制定TFR为治疗目标，合理应用TKI以及新型BCR::ABL1激酶抑制剂阿思尼布的加入，我们展望将来有更高比例的CML患者能达到TFR，终身摆脱治疗，避免药物长期不良反应，从而获得长久治愈。
